# Schisandrin A protects intestinal epithelial cells from deoxynivalenol-induced cytotoxicity, oxidative damage and inflammation

**DOI:** 10.1038/s41598-019-55821-4

**Published:** 2019-12-16

**Authors:** Murphy L. Y. Wan, Paul C. Turner, Vanessa A. Co, M. F. Wang, Khaled M. A. Amiri, Hani El-Nezami

**Affiliations:** 1 School of Biological Sciences, Faculty of Science, Kadoorie Biological Sciences Building, The University of Hong Kong, Pokfulam, Hong Kong; 2 Maryland Institute for Applied Environmental Health, School of Public Health, University of Maryland, College Park, Maryland, USA; 3 College of Science, Biology Department, United Arab Emirates University, Al Ain, United Arab Emirates; 4 Institute of Public Health and Clinical Nutrition, University of Eastern Finland, Kuopio, Finland

**Keywords:** Nutrient signalling, Mechanisms of disease

## Abstract

Extensive research has revealed the association of continued oxidative stress with chronic inflammation, which could subsequently affect many different chronic diseases. The mycotoxin deoxynivalenol (DON) frequently contaminates cereals crops worldwide, and are a public health concern since DON ingestion may result in persistent intestinal inflammation. There has also been considerable attention over the potential of DON to provoke oxidative stress. In this study, the cytoprotective effect of Schisandrin A (Sch A), one of the most abundant active dibenzocyclooctadiene lignans in the fruit of *Schisandra chinensis* (Turcz.) Baill (also known as Chinese magnolia-vine), was investigated in HT-29 cells against DON-induced cytotoxicity, oxidative stress and inflammation. Sch A appeared to protect against DON-induced cytotoxicity in HT-29 cells, and significantly lessened the DON-stimulated intracellular reactive oxygen species and nitrogen oxidative species production. Furthermore, Sch A lowered DON-induced catalase, superoxide dismutase and glutathione peroxidase antioxidant enzyme activities but maintains glutathione S transferase activity and glutathione levels. Mechanistic studies suggest that Sch A reduced DON-induced oxidative stress by down-regulating heme oxygenase-1 expression via nuclear factor (erythroid-derived 2)-like 2 signalling pathway. In addition, Sch A decreased the DON-induced cyclooxygenase-2 expression and prostaglandin E2 production and pro-inflammatory cytokine interleukin 8 expression and secretion. This may be mediated by preventing DON-induced translocation of nuclear factor-κB, as well as activation of mitogen-activated protein kinases pathways. In the light of these findings, we concluded that Sch A exerted a cytoprotective role in DON-induced toxicity *in vitro*, and it would be valuable to examine *in vivo* effects.

## Introduction

Oxidative stress occurs when there is an imbalance between cellular reactive oxygen species (ROS) and the antioxidant capacity^1^. ROS production is an essential physiological step involved in inflammation and tissue repair. It represents part of the defence mechanisms against intruding microorganisms, as well as of tissue healing and remodelling. Inflammation, together with oxidative stress could result in many pathological conditions, such as chronic intestinal inflammatory diseases.

Mycotoxins are noxious fungi-producing secondary metabolites that frequently contaminate food and feed, which raise great concern in relation to cancer, immune-suppression and growth retardation. Mycotoxins has estimated to contaminate about 25% of the world’s crops, and hence these naturally occurring toxins cause both economic losses and pose a substantial global public health risk. The trichothecene toxin deoxynivalenol (DON), though is not the most lethal mycotoxin, causes a significant food safety issues due to its high prevalence in cereals as reported by European Union Member States^2^. In a recent scientific report from EFSA^3^, it was shown that DON was detected in almost half of the food and feed products, including wheat, maize and oat grains and derived products. The total population are in general exposed to DON at levels below the daily Tolerable Daily Intake (TDI) of 1 μg/kg body weight (b.w.)^4^, despite sometimes approaching to it, especially in youn children. Children were found to expose to on average between 0.54 and 1.02 μg/kg b.w. of DON per day (upper bound) and between 0.95 and 1.86 μg/kg b.w. of DON per day (95^th^ percentile).

It has been reported that the symptoms of DON ingestion in humans include nausea, vomiting, diarrhea, stomachache, headache, dizziness, and fever^5^. The intestinal layer, is as a frontline of defence system against contaminants which acts by regulating the immune response to these intrusions. DON has previously been shown to compromise the gastrointestinal physical barrier^6–12^ and alter mucus production^10,13,14^ by many previous studies. Recently it is speculated that ingestion of certain mycotoxins including DON can lead to induction and/or persistence of human chronic intestinal inflammatory diseases in susceptible populations^15^.

DON triggers epithelial inflammation and systemic inflammation, by stimulating pro-inflammatory cytokines production in different epithelial cells and immune cells^16^. Acute DON ingestion perturbs the gastrointestinal tract in a way similar to symptoms of inflammatory bowel disease (IBD), therefore it is assumed that DON might contribute to IBD by dysregulating inflammatory process in the gut^17,18^. Whether or not DON consumption can induce intestinal inflammation were investigated using intestinal cell models in few previous studies. For example, Van De Walle *et al*.^19,20^ found that high DON intakes could cause or aggravate intestinal inflammation by activating nuclear factor κB (NF-κB) and stimulating pro-inflammatory cytokine release in intestinal epithelial cells. Recently, it was reported that DON led to inflammation and exacerbate inflammatory response in non-cancerous intestinal epithelial cells (IEC-6) following exposure to lipopolysaccharide (LPS) and interferon (IFN)γ, by increasing production of tumor necrosis factor (TNF)α, formation of nitrotyrosine, release of ROS, up-regulating inducible nitric oxide synthase (iNOS) and cyclooxygenase-2 (COX-2) expression, as well as activating NF-κB, nuclear factor (erythroid-derived 2)-like 2 (Nrf2) and inflammasome^21^. Our previous laboratory data has also revealed that physiologically relevant concentrations of DON can increase several pro-inflammatory cytokine mRNA levels in a porcine jejunal epithelial cell line^22^. Taken together, this suggests that DON could cause persistent intestinal inflammation and this may predispose to various intestinal inflammatory diseases including IBD.

Over the past several years, there has also been increasing attention over the potential of DON to induce oxidative stress. A recent review by Mishra *et al*. 2014 have summarised various cell culture and animal studies that demonstrated the potential involvement of oxidative stress in DON toxicity^23^. Among these studies, several studies conducted on intestinal cell lines have shown DON-induced cytotoxicity and apoptosis via oxidative stress mechanisms^24–28^. DON-dependent production of ROS may cause damage to proteins, lipids and DNA^23^. Since DON frequently occurs in food and feed, further studies on the relation of this toxin to oxidative stress and inflammation on intestinal cells, and the effective measures to alleviate its toxicity are needed.

Evidence gathered over the last decade suggests the potential health benefit of polyphenols, which are abundent in numerous plant-based foods and drinks, with daily consumption estimated at up to several hundred milligrams^29,30^. Most polyphenols exhibit antioxidant properties which can help protect against various oxidative stress relating diseases/disorders, such as cardiovascular diseases, cancers, inflammation, etc^31^. While systemic availability of ingested polyphenols is low^32^, gut enterocytes are continuously exposed to relatively high levels. Moreover, polyphenols are abundant antioxidants found in the lower gut^32^. As such, intake of polyphenols can produce both local and systemic effects in the gut. In addition to their antioxidant properties, studies have also suggested the benefits of dietary intake of polyphenols for their anti-inflammatory functions due to their ability to affect different inflammation-related biomarkers such as pro-inflammatory cytokines^33^.

The fruit of *Schisandra chinensis* (Turcz.) Baill has widely been used in Far Eastern medicine for treating various diseases, including gastrointestinal illnesses^34^. The major bioactive constituents found in *Schisandra chinensis* (Turcz.) Baill are dibenzocycliooctadiene derivative lignans^35^. Among them, Schisandrin A (Sch A) is one of the most abundant active dibenzocyclooctadiene lignans, and has been reported to inhibit inflammation and reduce free radicals. For example, Sch A considerably decreased cell apoptosis and necrosis, increased cell survival and reduced lactate dehydrogenase (LDH) release in oxygen glucose deprivation/reperfusion-induced cell injury in primary culture of rat cortical neurons^36^. Moreover, Sch A has shown to protect human neuroblastoma SH-SY5Y cells from serum and glucose deprivation injury, possibly through the regulation of inflammation and cell apoptosis through modulating nucleotide-binding domain and leucine-rich repeat containing protein 3 (NLRP3)/NF-кB/Caspase-1/interleukin 1β (IL1β), c-Jun N-terminal kinase (JNK)/mitogen-activated protein kinase (MAPK), and caspase-3 signalling pathways^37^. Sch A also significantly reduced the production of nitric oxide (NO), TNFα and interleukin 6 (IL6) stimulated by LPS in microglial cells, by inhibiting the TNF receptor-associated factor (TRAF6)- inhibitor of nuclear factor kappa-B kinase (IKKβ)-NF-κB and Janus kinase 2 (Jak2)-signal transducer and activator of transcription 3 (Stat3) signalling pathways^38^. Similarly, Sch A was demonstrated to exhibit anti-inflammatory action in LPS-stimulated RAW264.7 macrophages, by inhibiting the pro-inflammatory NK/p38 kinase/NF-κB signalling pathway and suppressing various pro-inflammatory mediators production. Sch A also decreased the cellular reduced glutathione (GSH) level and increased glutathione S-transferase activity, implying the causal relationship between the anti-inflammatory action and antioxidant effects^39^. Recently, a study by Kwon *et al*. has also reported that Sch A remarkably reduced the LPS-induced inflammatory and oxidative responses by down-regulating the NF-κB, MAPK and phosphoinositide 3-kinase (PI3K)/Akt pathways; these effects are mediated partly by the activation of the Nrf2/heme oxygenase-1 (HO-1) pathway^40^. Despite the protective role of Sch A has been reported in the different aspects stated above, the underlying molecular mechanisms regarding the anti-inflammatory and antioxidant capacity of Sch A have yet to be fully revealed. Also, Sch A has never been investigated before for its ability against DON-induced oxidative stress, inflammation and apoptosis. Accordingly, we hypothesized that Sch A can protect against DON-induced oxidative stress, inflammation and apoptosis on intestinal epithelial cells, which suggests a possible protective role in preventing or delaying the progression of chronic intestinal inflammatory diseases, such as IBD. This study aimed to test the impact of Sch A on DON induced oxidative stress, inflammation and apoptosis on a HT-29 human intestinal cell line. The underlying molecular mechanisms were also examined. HT-29 cells, though was originated from a human colorectal carcinoma, was selected as the intestinal model *in vitro*, and it was also used in few other previous studies^27,28^.

## Results

### Sch A protects against DON-induced cytotoxicity in HT-29 cells

**Figure 1:**
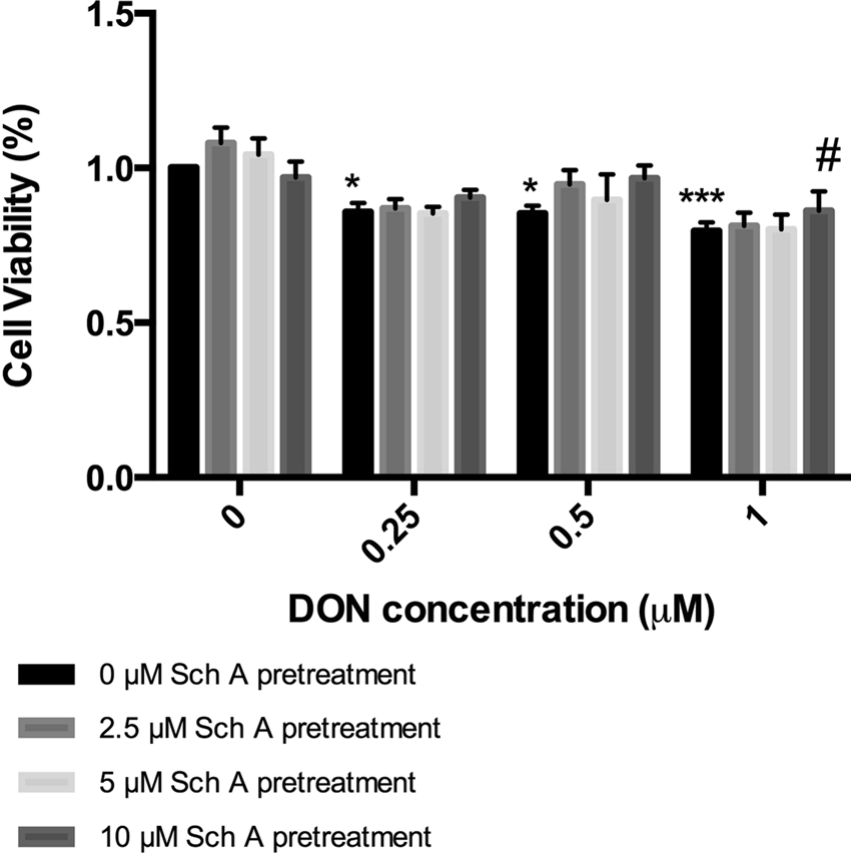
Sch A protects DON-induced toxicity in HT-29 cells by maintaining cell viability. HT-29 cells were treated with DON (0.25, 0.5 and 1 µM) for 24 hours in the presence and absence of Sch A pre-treatment (2.5, 5 and 10 µM) for 24 hours. Control received appropriate carriers. Results were shown as mean of ±SEM (n ≥ 6), which are at least six separate experiments performed in six replicates. *^,^ ***p < 0.05 and 0.001 significantly different from control, ^#^*p* < 0.05 significantly different from DON treated cells.

To determine cell viability and Sch A and DON concentrations used for subsequent studies, CCK assay was performed (Fig. 1). All the tested DON concentrations caused a significant decrease in cell viability compared to control (*p* < 0.001). DON treatment with 10 µM Sch A pre-treatment significantly protected the cells at 1 µM DON concentration (*p* < 0.05), but the curative effect was insignificant at 2.5 and 5 µM concentrations of Sch A. No significant curative effect was observed for 0.25 and 0.5 µM DON concentration with any Sch A concentrations pre-treatment, which might be attributed to the large deviations within groups. Sch A treatment alone did not change the cell viability. Since Sch A caused a better cytoprotective effect at 1 µM DON concentration, further experiments were carried out with different concentrations of Sch A (2.5–10 µM) pre-treatment followed by DON exposure at 1 µM.

### Sch A prevents DON-induced cell cycle arrest and, to a lesser extent, apoptosis

**Figure 2:**
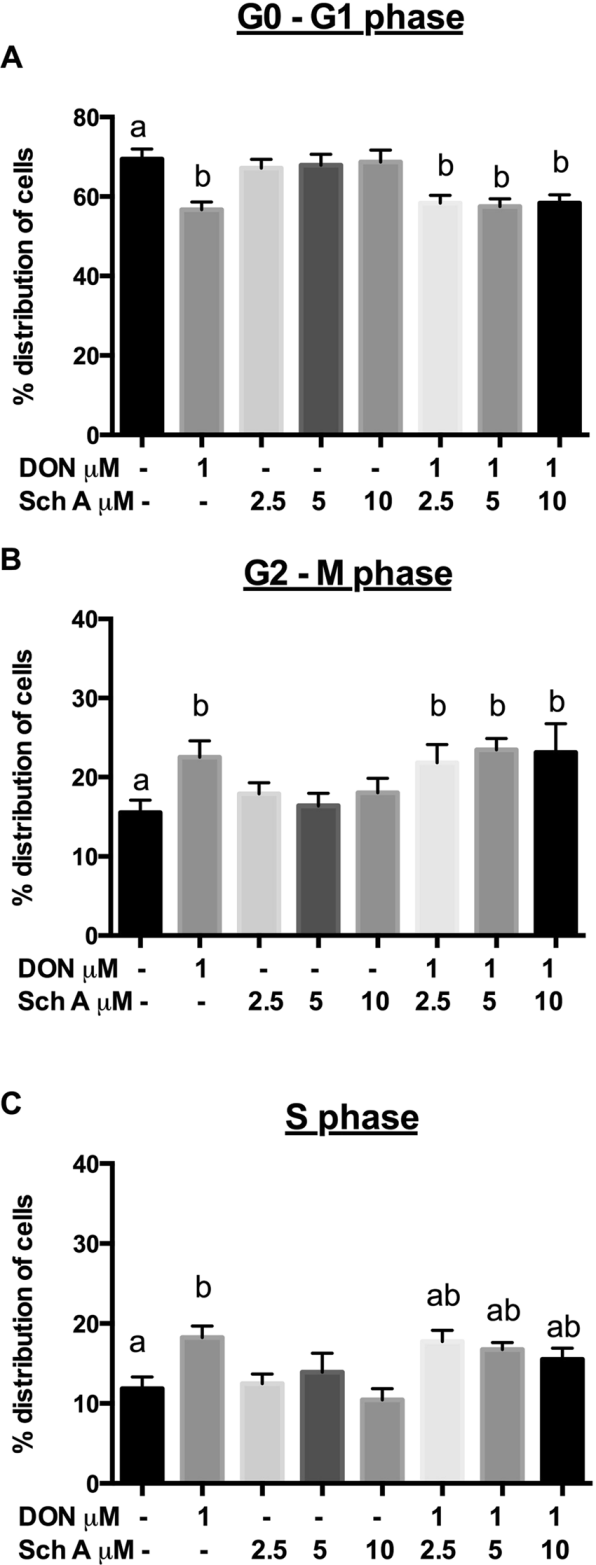
Sch A tends to protect DON-induced cell cycle arrest by reducing the percentages of cells in S phases. HT-29 cells were treated with 1 µM of DON for 24 hours in the presence and absence of Sch A pre-treatment (2.5, 5 and 10 µM) for 24 hours. Control received appropriate carriers. Percentage distribution of cells in (**A**) G0-G1 phases, (**B**) G2-M phases and (**C**) S phases were shown as mean of ±SEM (n ≥ 6), which are at least six separate experiments. Different letters indicate significant differences at *p* < 0.05. G0, resting phases; G1, growth 1 phase; G2, growth 2 phase; M phase, mitosis; S phase, synthesis phase.

Since the induction of apoptosis may be mediated through the regulation of cell cycle, the impact of different treatments on cell cycle perturbations was analysed by PI staining (Fig. 2). DON alone showed significant decrease in the percentage distribution of cells in G0-G1 phase (Fig. 2A). Percentages of cells in S phase (Fig. 2B) and G2-M phase (Fig. 2C) were significantly increased following DON treatment alone. These data indicate that DON alone induced S-phase and G2-M phase cell cycle arrest. Pre-treatment with Sch A, tends to decrease the percentages of cells in S phases, but significant differences were not detected. No significant changes in percentages of cells in G0-G1, S- and G2-M phases occurred when cells were treated with any of Sch A concentrations alone (Fig. 2).

**Figure 3:**
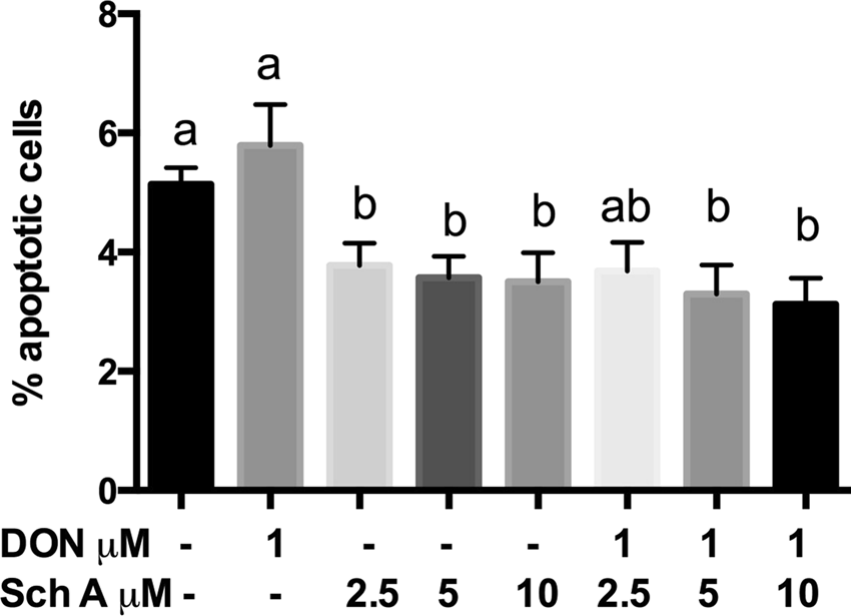
Sch A pre-treatment reduced the proportion of apoptotic cells compared to DON-treated cells. HT-29 cells were treated with 1 µM of DON for 24 hours in the presence and absence of Sch A pre-treatment (2.5, 5 and 10 µM) for 24 hours. Control received appropriate carriers. Annexin V fluorescein isothiocyanate (FITC)- or propodium iodide (PI)-positive cells were counted as apoptotic cells, and the remaining cells were designated the surviving cell fraction. Proportions of apoptotic cells were shown as mean of ±SEM (n ≥ 6), which are at least six separate experiments. Different letters indicate significant differences at *p* < 0.05.

For quantification of apoptosis, cells were stained with Annexin V-FITC and PI. Both early and late apoptotic cells were considered apoptotic cells. In Fig. 3, apoptosis induced by DON was not significantly higher compared to control cells, whereas Sch A treatment alone resulted in significant reduction in apoptosis at all the concentrations tested. This may be due to the relatively low concentration of DON used in this study, which may be insufficient to induce apoptosis. However, Sch A pre-treatment at 5 and 10 µM showed significant reductions in the proportion of apoptotic cells compared to DON-treated cells.

### Sch A inhibits DON-induced ROS production, nitrite production but not lipid peroxidation

**Figure 4:**
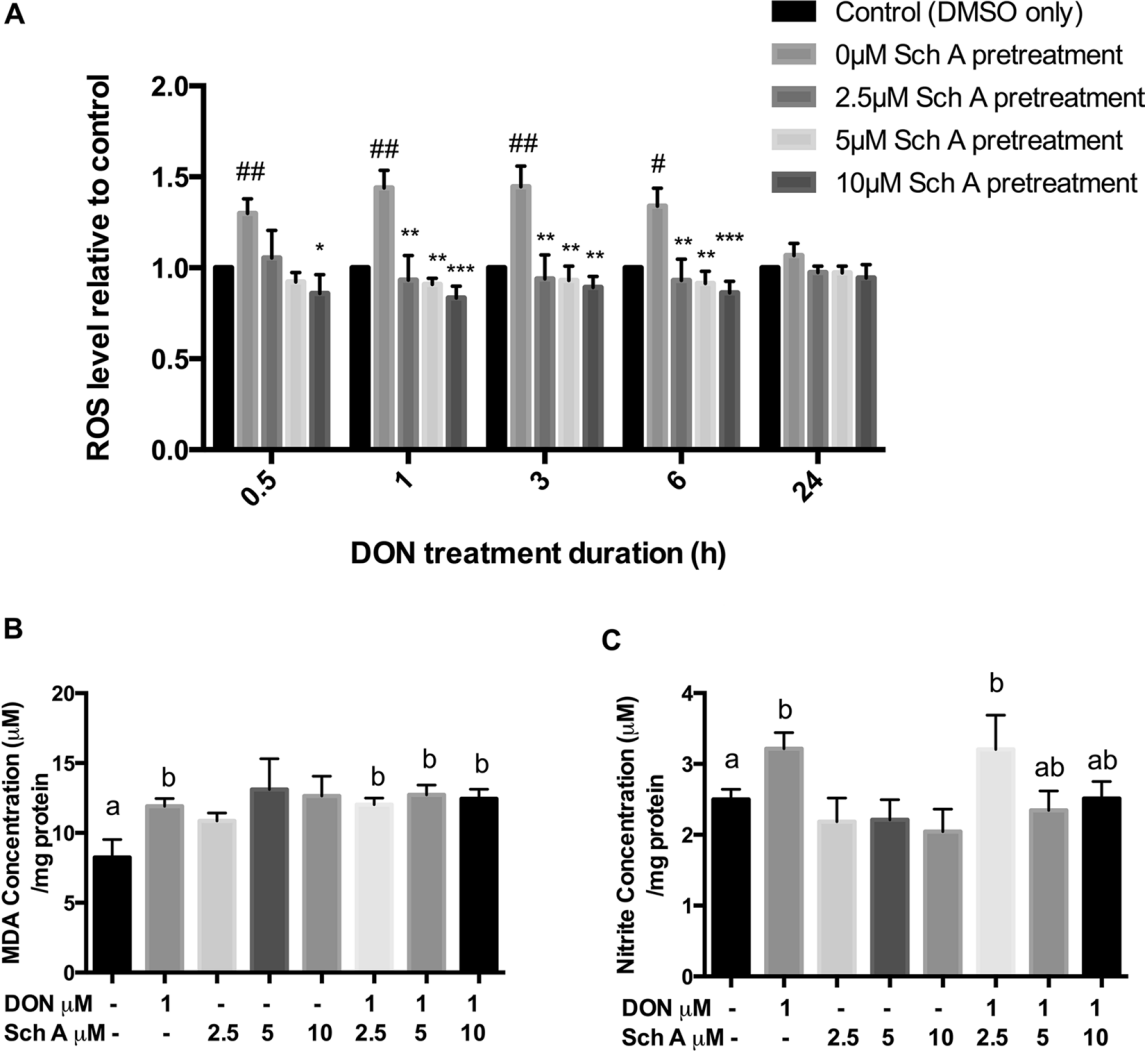
Sch A inhibits DON-induced reactive oxidative species (ROS) generation, nitrite production but not lipid peroxidation. (**A**) HT-29 cells were treated with fluorescent dye DCF-DA, to measure DON-induced intracellular ROS generation by fluorimetry. Cells were treated with 1 µM of DON for 0.5–6 hours following DCF-DA treatment for 30 minutes. For Sch A pre-treatment, cells were exposed to Sch A (2.5, 5 and 10 µM) for 24 hours, then exposed to DCF-DA for 30 minutes followed by 1 µM of DON for 0.5–6 hours. For cells with 24 hours of DON treatment, DCF-DA was added at the end of the incubation. Results were shown as mean of ±SEM (n ≥ 6), which are at least six separate experiments performed in triplicates. ^#, ##^*p* < 0.05 and 0.01 significantly different from control, *^,^**^,^***p < 0.05, 0.01 and 0.001 significantly different from DON treated cells. (**B**) Nitrite generation measured using Griess reagent. (**C**) Malondialdehyde (MDA) prodiction as measured by thiobarbituric acid reactive substances (TBARS) assay. Cells were treated with 1 µM of DON for 24 hours in the presence and absence of Sch A pre-treatment (2.5, 5 and 10 µM) for 24 hours. Results were shown as mean of ±SEM (n ≥ 6), which are at least six separate experiments. Different letters indicate significant differences at *p* < 0.05.

To measure changes in the cellular redox status in response to DON with or without Sch A pre-treatment, cells were first exposed to 2.5–10 µM Sch A followed by DON exposure at 1 µM from 0.5 to 24 hours (Fig. 4A). Treatment with DON significantly increased ROS from 0.5 to 6 hours (*p* < 0.05) but not at 24 hours. Pre-treatment with Sch A at 2.5, 5 and 10 µM followed by DON caused a significant decline in ROS release (*p* < 0.05).

Thiobarbituric acid reactive substance (TBARS) assay was carried out to measure MDA production in HT29 cells (Fig. 4B). Results obtained demonstrated that DON increased MDA level significantly when compared to control (*p* < 0.05), while Sch A alone did not result in significant change. MDA production in samples pre-treated Sch A remained comparable to that of DON alone.

NO assay was performed to measure effects of DON and Sch A on nitrite production (Fig. 4C). Treatment with DON significantly reduced nitrite concentration (*p* < 0.05) but pre-treatment with 5 µM and 10 µM Sch A increased nitrite production slightly, though not statistically significant compared to samples treated with DON only. Samples treated with Sch A only did not have a significantly different nitrite concentration than control.

### Sch A lowers DON-induced CAT, SOD and GPx antioxidant enzyme activities but maintains GST activity and GSH levels

**Figure 5:**
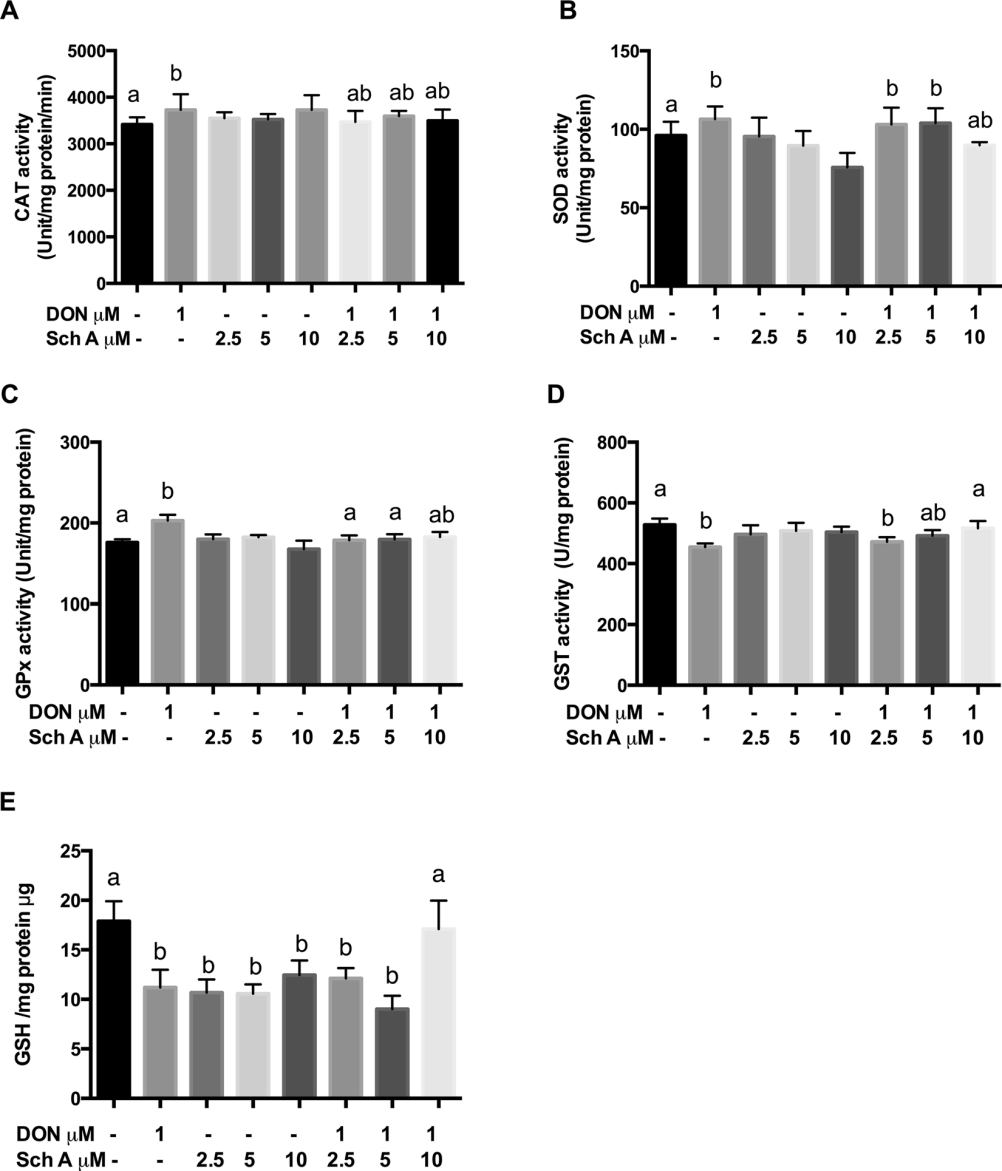
Sch A lowers DON-induced catalase (CAT), superoxide dismutase (SOD) and glutathione peroxidase (GPx) antioxidant enzyme activities but maintains GST activity and GSH levels. HT-29 cells were treated with 1 µM of DON for 24 hours in the presence and absence of Sch A pre-treatment (2.5, 5 and 10 µM) for 24 hours. The cell lysates were analysed for (**A**) CAT, (**B**) SOD, (**C**) GPx, (**D**) GST activities and (**E**) GSH levels. Catalase 1U = The amount of enzyme that consumes 1 nmoles H_2_O_2_/minute, GST 1U = The amount of enzyme that conjugates 1 µM CDNB/minute, GPx 1U = The amount of enzyme that converts 1 µM GSH to GSSG in the presence of H_2_O_2_/minute. SOD 1U = The amount of enzyme required to give 50% inhibition of pyragallol auto-oxidation. Results were shown as mean of ± SEM (n ≥ 6), which are at least six separate experiments. Different letters indicate significant differences at *p* < 0.05.

CAT, SOD, GPx activity assays were performed to examine how Sch A and DON affected antioxidant enzyme activities (Fig. 5A–C). CAT, SOD and GPx are primary enzymes participated in repairing damage caused by oxidative stress. DON alone increased the activities of these enzymes significantly (*p* < 0.05). But Sch A pre-treatment showed no significant changes in CAT activity and SOD activity, while the decrease in GPx activity was statistically significant (*p* < 0.05). Sch A alone slightly increased CAT activity but did not affect other enzyme activities significantly.

GST are detoxification enzymes that act through conjugation of xenobiotics and GSH, an antioxidant that protects cells from ROS. DON significantly reduced GST activity and GSH level (*p* < 0.05), but pre-treatment with 10 µM Sch A increased GST activity and GSH level significantly (*p* < 0.05) when compared with samples treated with DON alone. It is noteworthy that Sch A alone significantly (*p* < 0.05) lowered GSH levels (Fig. D,E).

### Sch A suppresses DON-induced HO-1 expression through modulation of Nrf2 signalling pathway

**Figure 6:**
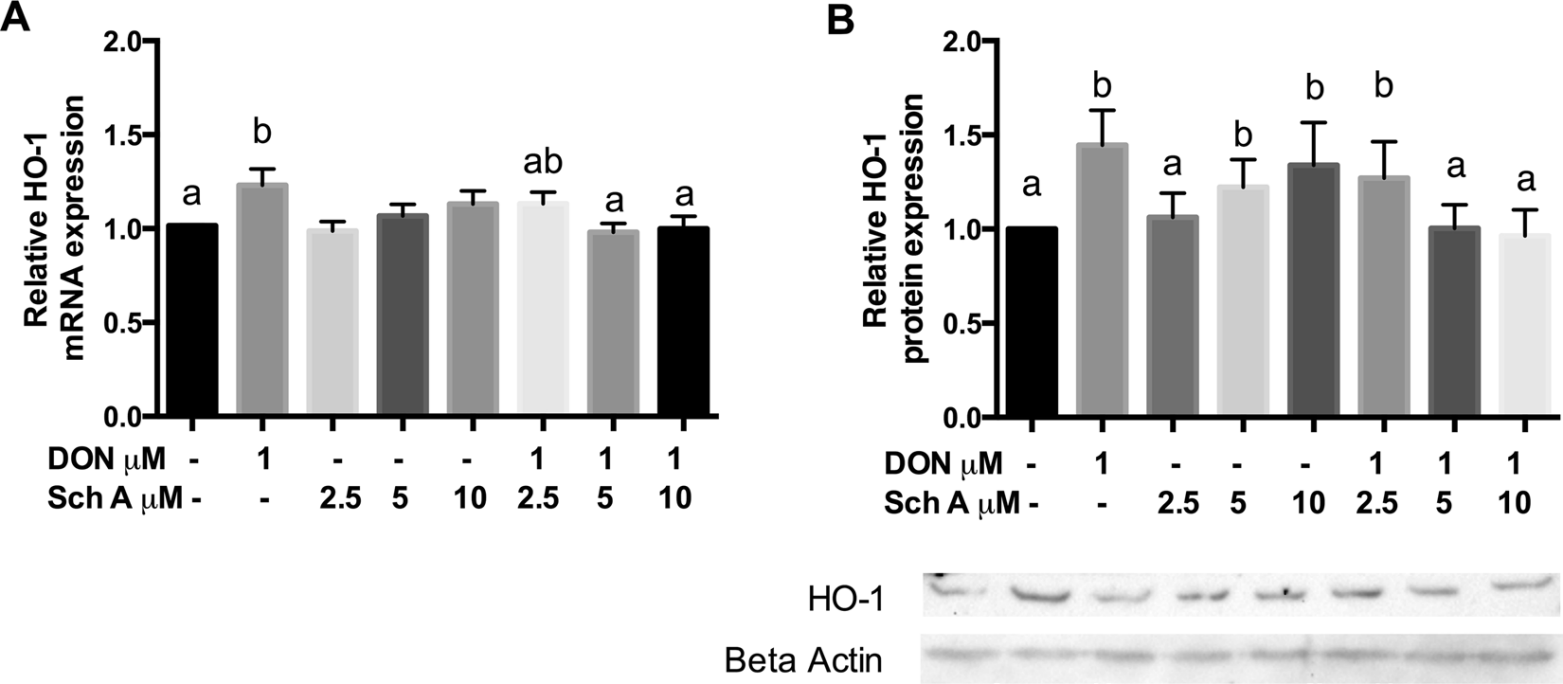
Sch A suppresses DON-induced heme oxygenase-1 (HO-1) mRNA and protein expression. HT-29 cells were treated with 1 µM of DON for 24 hours in the presence and absence of Sch A pre-treatment (2.5, 5 and 10 µM) for 24 hours. (**A**) HO-1 mRNA expression was measured by qPCR, with GAPDH as the internal control; (**B**) HO-1 protein expression was measured by Western blotting, with beta actin as the internal control. (**C**) Representative photos of western blotting of HO-1 and beta actin. Results were shown as mean of ± SEM (n ≥ 6), which are at least six separate experiments. Different letters indicate significant differences at *p* < 0.05.

The HO-1 mRNA and protein expression in HT-29 cells were displayed in Fig. 6. DON significantly up-regulated HO-1 mRNA and protein expression level. With Sch A pre-treatment, both HO-1 mRNA and protein were suppressed. Sch A alone caused significant up-regulation of HO-1 protein levels but not mRNA levels.

**Figure 7:**
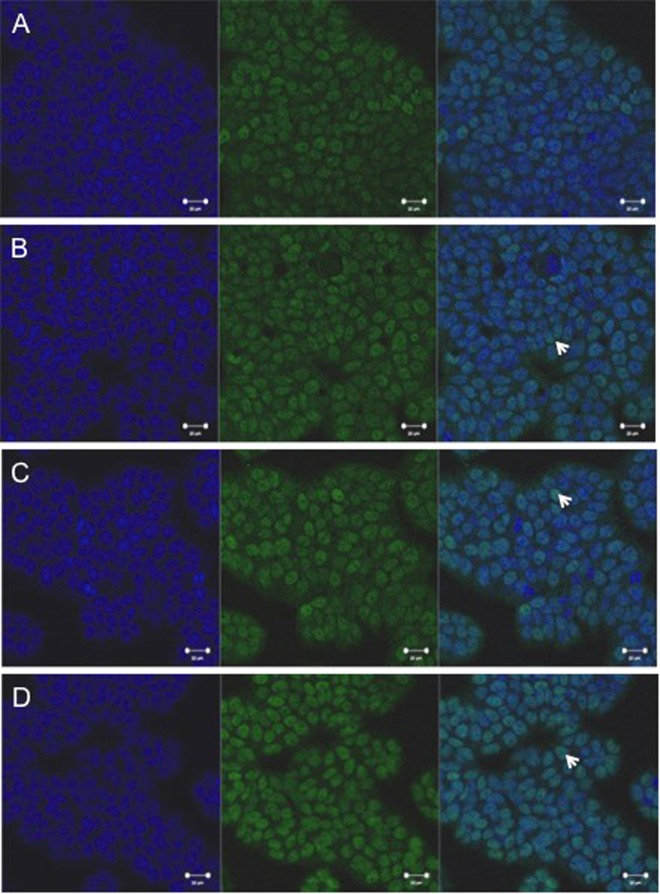
Sch A activated nuclear factor (erythroid-derived 2)-like 2 (Nrf2) signalling pathway. HT-29 cells were treated with 1 µM of DON for 3 hours, in the presence and absence of Sch A pre-treatment (10 µM for 24 hours), fixed and studied for localization of Nrf2 through indirect immunofluorescence using Alexa Fluor® 488 secondary antibody. The nucleus was stained with Hoechst staining. Figure shows Hoechst staining (blue), Alexa Fluor® 488 (green), and overlay. All photos shown were taken at 400x magnification. (**A**) Control cells showing Nrf2 retention in the cytoplasms. (**B**) Cells exposed to DON for 3 hours showing that Nrf2 has migrated into the nucleus. (**C**) Cells pre-treated with Sch A for 24 hours followed by DON exposure for 3 hours, showing increased Nrf2 translocation to the nucleus. (**D**) Cells treated with Sch A alone, showing the greatest Nrf2 translocation in the nucleus. Arrows indicate cells with Nrf2 translocation in the nucleus.

To examine the effect of DON and Sch A on the activation of Nrf2, Nrf2 was labelled with a green fluorescent tag. As revealed in Fig. 7, nuclear staining of Nrf2 was increased after three hours when DON was added. Pre-treatment with Sch A also increased nuclear Nrf2, indicating an activation of Nrf2 signalling pathway.

### Sch A suppresses DON-induced COX-2 mRNA and protein expression and PGE2 production

**Figure 8:**
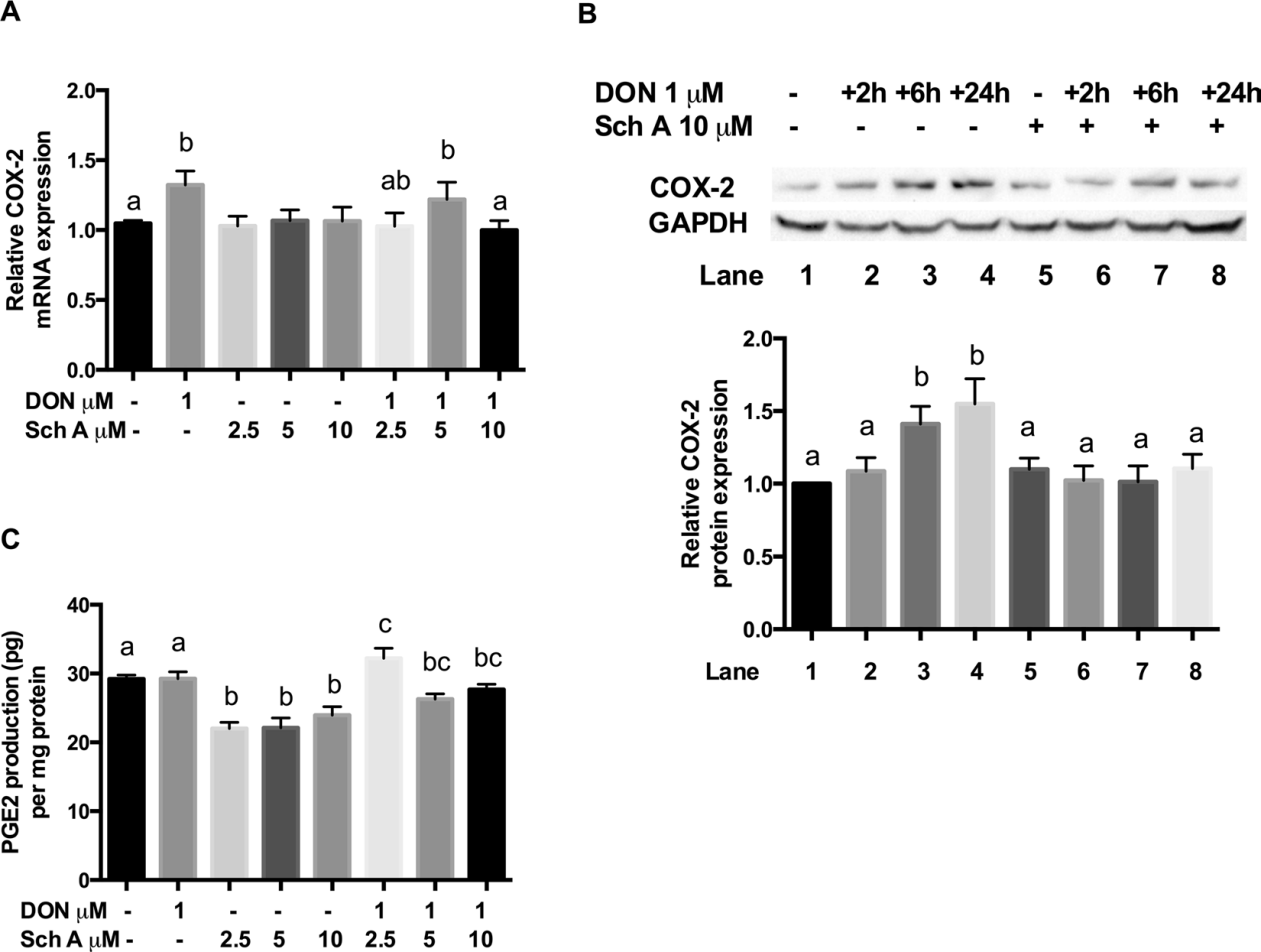
Sch A suppressed DON-induced cyclooxygenase-2 (COX-2) mRNA and protein expression and prostaglandin E2 (PGE2) production. HT-29 cells were treated with 1 µM of DON for different time periods as indicated in the fig. in the presence and absence of Sch A pre-treatment (2.5, 5 and 10 µM) for 24 hours. (**A**) COX-2 mRNA expression was measured by qPCR, with GAPDH as the internal control; (**B**) COX-2 protein expression was measured by Western blotting, with GAPDH as the internal control, and representative photos of western blotting of COX-2 and GAPDH; (**C**) PGE2 production was determined by ELISA. Results were shown as mean of ±SEM (n ≥ 6), which are at least six separate experiments. Different letters indicate significant differences at *p* < 0.05.

Cyclooxygenase (COX)-2 is regarded as an inflammation marker. mRNA and protein expression of COX-2 in DON treated cells without or with Sch A pre-treatment were measured. Figure 8A indicated that DON treatment significantly increased mRNA expression. Pre-treatment of Sch A down-regulated the COX-2 mRNA significantly. Sch A alone did not cause any changes in the COX-2 mRNA expression. Previous reports indicate that COX-2 is not solely regulated at the transcription level but also via post-transcriptional mechanisms in human intestinal cells. Moreover, COX-2 protein was degraded through ubiquitin proteolysis, and its half-life was ∼3.5–8 h^41^. Therefore, protein expression of COX-2 was measured in cells after 2, 6 and 24 hours of exposure to DON. Western blot analyses (Fig. 8B) showed that DON significantly up-regulated COX-2 protein expression after 6 and 24 hours of DON exposure. Sch A pre-treatment significantly down-regulated COX-2 protein expression after 6 and 24 hours of exposure to DON.

PGE2 is one of the important mediators produced at the inflammatory sites by the COX-2 enzyme. Thus, the impact of Sch A on DON-induced production of PGE2 in HT-29 cells was also studied by quantifying its levels in the cellular supernatant using ELISA. Figure 8C indicates that stimulation of HT-29 cells with DON alone did not increase PGE2 concentration in the culture medium; however, with Sch A pre-treatment, PGE2 production was significantly reduced. Sch A alone significantly decreased PGE2 production compared to control.

### Sch A prevents DON-induced NF-κB expression and nuclear localization of NF-κB

**Figure 9:**
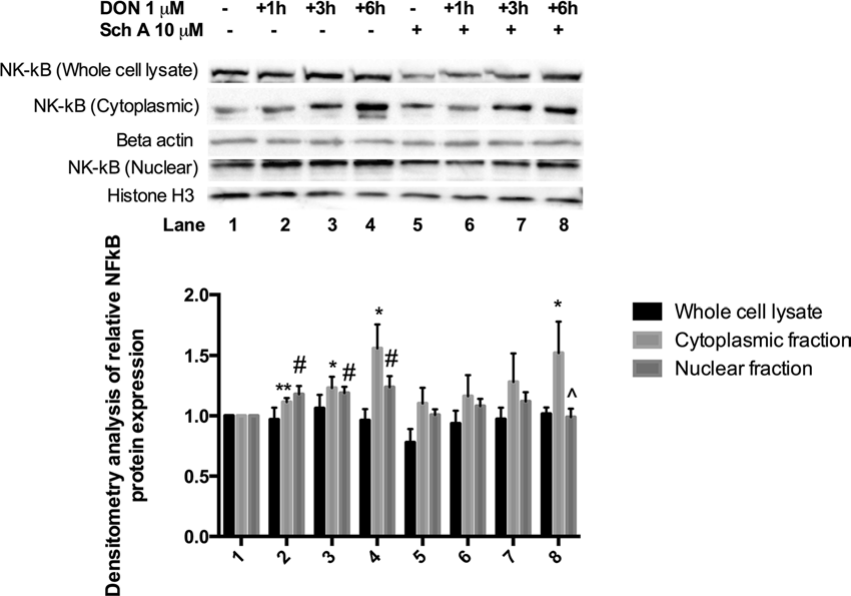
Sch A prevents DON-induced nuclear factor κB (NF-κB) expression and nuclear localization of NF-κB. HT-29 cells were treated with 1 µM of DON for different time periods as indicated in the fig. in the presence and absence of Sch A pre-treatment (2.5, 5 and 10 µM) for 24 hours. Samples were prepared and subjected to Western blot analysis as described in materials and methods. Western blot showing time course for NF-κB expression. Pre-treatment with Sch A significantly decreased cytoplasmic and nuclear NF-κB expression. Densitometry analysis of NF-κB Western blots, statistical analysis was carried out by student’s t test. Results were shown as mean of ±SEM (n ≥ 6), which are at least six separate experiments. *^,^ **p < 0.05, 0.01 compared to control. ^#^*p* < 0.05 com*p*ared to control. ^*p* < 0.05 compared to DON-treated samples.

Western blot analyses were performed at various time intervals of 1, 3 and 6 hours for NF-κB expression (Fig. 9). The results indicate that not significant changes in NF-κB protein expression were observed in the whole cell extracts of DON-treated cells. We then examined the NF-κB protein expression in both cytoplasmic and nuclear fractions. Significant up-regulation of NF-κB protein expression was observed in the cytoplasmic fractions of DON-treated cells at all time points. However, pre-treatment of Sch A did not suppress NF-κB protein expression. In nuclear fractions, DON significantly up-regulated NF-κB protein expression at all time points; only pre-treatment with Sch A at 10 µM significantly down-regulated NF-κB protein expression. The results indicate that Sch A affected DON-induced NF-κB expression by preventing nuclear localization of NF-κB.

### Sch A inhibits DON-induced production of pro-inflammatory cytokine IL8

**Figure 10:**
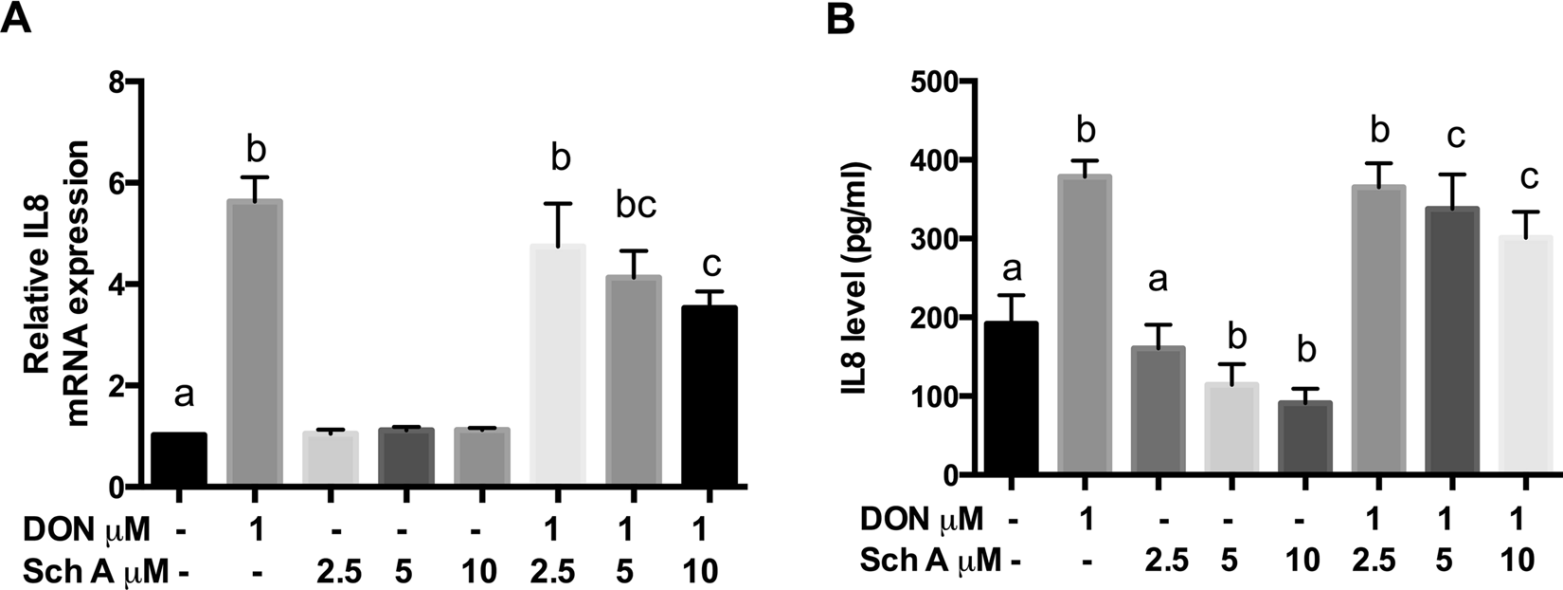
Sch A suppressed DON-induced interleukin 8 (IL8) mRNA and secretion. HT-29 cells were treated with 1 µM of DON for 24 hours in the presence and absence of Sch A pre-treatment (2.5, 5 and 10 µM) for 24 hours. (**A**) IL8 mRNA expression was measured by qPCR, with GAPDH as the internal control; (**B**) Amount of IL8 secreted in the cell supernatant was measured by ELISA. Results were shown as mean of ±SEM (n ≥ 6), which are at least six separate experiments. Different letters indicate significant differences at *p* < 0.05.

We then assessed if the Sch A effects on NF-κB activation were followed by an effect on IL8 mRNA expression and secretion (Fig. 10). DON treatment resulted in significant up-regulation of IL8 mRNA and amount of IL8 released in the medium as quantified by qPCR and ELISA, respectively. Sch A pre-treatment down-regulated IL8 mRNA and secretion in a concentration-dependent manner. It is also interesting to note that Sch A alone at 5 and 10 µM significantly reduced the amount of IL8 released but down-regulation of IL8 mRNA was not observed with the same treatments.

### Sch A inhibits DON-induced activation of MAPK pathways

**Figure 11:**
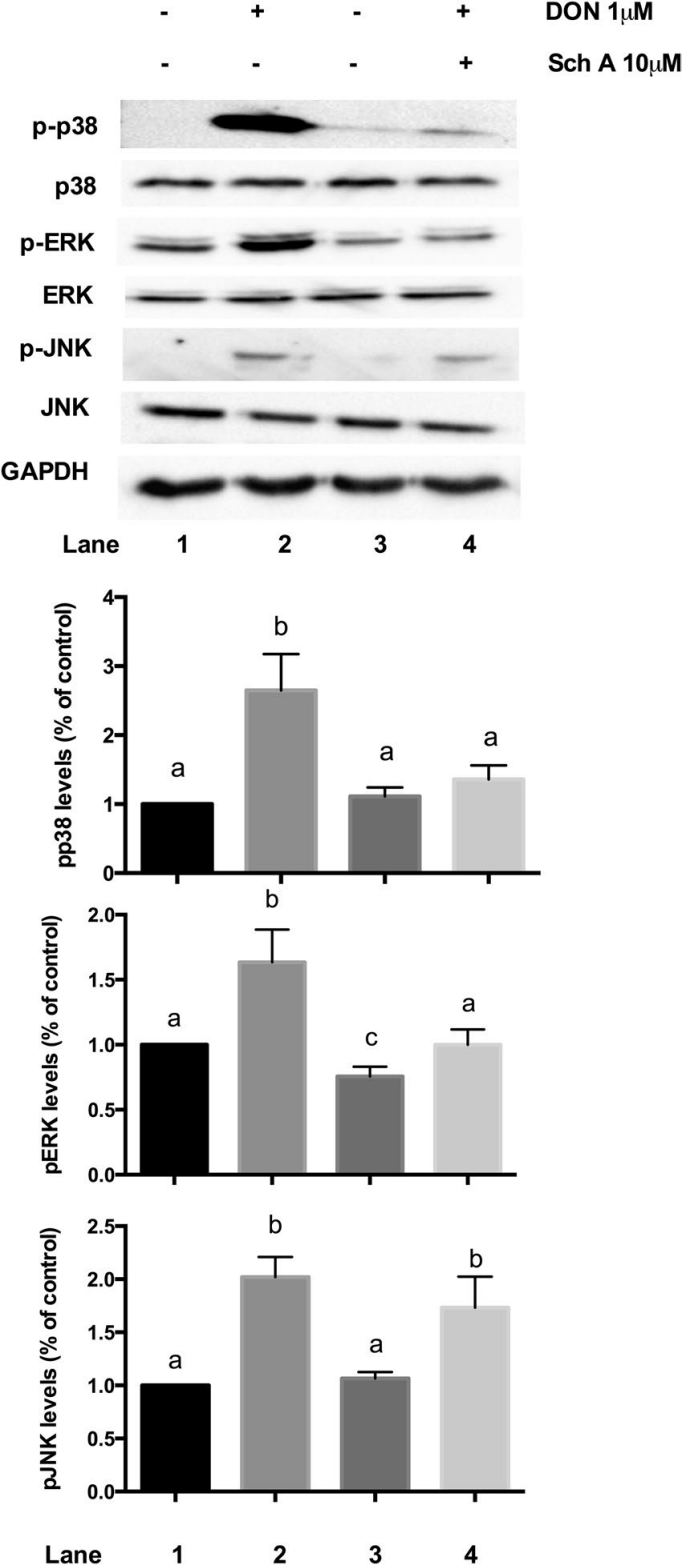
Sch A inhibits DON-induced activation of mitogen-activated protein kinase (MAPK) pathways. HT-29 cells were treated with 1 µM of DON for 24 hours in the presence and absence of Sch A pre-treatment (2.5, 5 and 10 µM) for 30 minutes. Protein samples were analysed by Western blot with phospho-specific antibodies. The total MAPK levels were used as an internal control. Representative photos of western blotting of MAPKs and GAPDH; desitometric analyses of immunoblots. Results were shown as mean of ±SEM (n ≥ 6), which are at least six separate experiments. Different letters indicate significant differences at *p* < 0.05.

Previous studies have established that the MAPK signalling pathways participated in DON-induced inflammation in intestinal epithelial cells^20,42^. Thus, the potential involvement of these signalling pathways in Sch A-mediated inhibition of DON-induced inflammation was investigated in this study. Western blot data showed that DON treatment markedly promoted the phosphorylation of three MAPKs, JNK, p38 MAPK and extracellular signal-regulated kinase (ERK) (Fig. 11); yet, the phosphorylation of p38 and ERK but not JNK were abrogated by pre-treatment with Sch A. Thus, it is likely that Sch A inhibited the inflammatory response by supressing p38 and ERK signalling pathways in DON-stimulated HT-29 intestinal cells.

## Discussion

DON is a ribotoxic mycotoxin that exerts its toxicity by provoking oxidative stress and inflammatory responses^27^. Oxidative stress causes many pathological changes, including inflammation and cancer. In principle, oxidative stress can result from increased ROS/reactive nitrogen species (RNS) production and lowered antioxidant defence. To combat oxidative stress and the molecular alterations associated with that, it is suggested that nutraceuticals or antioxidants may be supplemented via diet. The cytotoxic effects of DON are well documented, however, studies to alleviate the toxicity are sparse. Recently, Sch A was shown to suppress inflammation and oxidative stress in LPS-treated RAW 264.7 macrophages^40^. This study was the first to investigate how Sch A affected DON induced cytotoxicity, reactive oxidative and nitrogen species production and inflammation on a human HT-29 intestinal cells.

The results clearly showed that Sch A can act as a cytoprotective agent against DON-induced cytotoxicity in HT-29 cells. CCK assay revealed that Sch A at 10 μM offered cytoprotection by protecting the cells against DON-induced cytotoxicity. The cytoprotective effect of Sch A against DON-induced toxicity was further evaluated by examining cell cycle distribution and apoptosis. Our results suggest that DON induced G2-M and S phase arrest without induction of apoptosis, possibly attributed to the relatively low but physiologically relevant concentration of DON employed in the current study^43^. Sch A seems to inhibit DON-induced S phase arrest and cause a remarkable decrease in the proportion of apoptotic cells, implying the cytoprotection effect was possibly mediated via cell cycle regulation, to a lesser extent, affecting cell apoptosis.

It was believed that cytoprotective effect of Sch A against DON-induced toxicity may be attributable to the capacity of Sch A to act as a powerful antioxidant and free radical scavenger^44^. In the present study, Sch A can reduce DON-induced ROS at a concentration of 10 μM, implying that Sch A’s antioxidant and free radical scavenging activity contributes an important action in cytoprotection. Also, ROS production in HT-29 cells exposed to DON increased from 0.5 to 6 hours, but a negligible increase in ROS production was found at 24 hours. This was in accordance with previous studies, which showed significant release of ROS following DON treatment at shorter exposure time (e.g. 15–60 minutes)^27,28^ but not for longer exposure time (e.g. 24 hours)^26^. All these studies indicated that differences in exposure times could affect ROS generation.

In addition to ROS production, NO production was also measured. It was demonstrated that DON-induced NO production was partially inhibited by Sch A. Excessive production of ROS/RNS could induce oxidative stress and cause damage to lipids, proteins or DNA, thus affecting the normal cellular functions^45^. Consistent with previous studies, DON induced lipid peroxidation on intestinal cells as measured by MDA formation^25,27^. This may alter membrane integrity, cellular redox signalling and antioxidant status of the cells^46^. However, Sch A pre-treatment did not decrease the MDA production caused by DON in our study, suggesting that the protective mechanism of action of Sch A does not seem to be related to lipid peroxidation.

Cellular response to oxidative stress is dependent of enzymatic and non-enzymatic anti-oxidant defences in the cell^27,28^. To fight against oxidative stress, a balance between of these factors needs to be attained. In the current study, both enzymatic (SOD, CAT, GPx and GST) and non-enzymatic antioxidants (reduced GSH) were measured. SOD detoxifies a superoxide anion to H_2_O_2_, CAT and GPx then convert H_2_O_2_ to water and oxygen. GST is a secondary enzyme which plays a function in glutathione metabolism^47^. Extensive evidence in literature has shown that host cells respond to oxidative stress by increasing these antioxidant enzyme activities^48^. Our results are in agreement with earlier reports which demonstrated that DON resulted in elevated antioxidant enzymes SOD, CAT and GPx in response to oxidative stress^27,28^. Although oxidized glutathione (GSSG) was not measured in this study, a concomitant drop in total GSH levels was seen after DON treatment, which may be explained by the fact that GSH is used as an antioxidant in order to cease oxidative reactions triggered by DON by scavenging toxic free radicals and thus contribute to the anti-oxidative effects^49^. In fact, aberrant GSH levels can undesirably disturb various cellular processes, such as mitochondrial function, homeostasis and death^50^. Pre-treatment with Sch A, as an antioxidant, defended the cells from ROS, by preserving GSH levels. It is interesting to note that Sch A alone diminished cellular GSH level, which was in agreement with another study using macrophages/lymphocytes in mice^39^. The anti-inflammation effect produced by Sch A may be linked to their ability to decrease cellular GSH, which may be partly associated with the modification of a redox-sensitive regulatory cysteine residue in NF-κB under the reduced cellular GSH content environments^51^.

To further characterize the mechanisms responsible for Sch A-mediated anti-oxidative functions, our study assessed whether Sch A influenced the DON-induced activation of Nrf2 pathway that modulates oxidative stress and inflammatory responses by controlling crucial antioxidant and detoxification enzyme genes via the antioxidant response element. DON treatment induced cellular oxidative stress, Nrf2 then dissociated from Keap-1 followed by its translocation to the nucleus. Different antioxidant enzymes (such as HO-1, GPx, etc.) increased to keep cells from oxidative injury. In the current study, DON significantly up-regulated HO-1 expression, a cytoprotective enzyme synthesized by Nrf2 activation, which was consistent with earlier reports^21,52^. Upon the Nrf2 nuclear translocation, it up-regulated HO-1 expression that helped to protect cells against oxidative stress. Furthermore, HO-1 level was also significantly increased by Sch A alone, which may imply that Sch A itself plays an crucial cytoprotective effect by removing ROS to conquer oxidative damage, inflammation and apoptosis^53,54^. It is surprising to note that pre-treatment with Sch A at 5 and 10 μM followed by DON exposure significantly decreased HO-1 expression. It suggested that activation of HO-1 can cause an initial rise in the cellular antioxidant status, but its continuous activation would reduce it. The HO-1 protective effect might be restricted to a relatively limited threshold of overexpression, and excessive HO-1 may even sensitize the cell to oxidative stress by releasing reactive iron^55^. Our results showed that DON aggravated the oxidative stress response that resulted in the unwanted up-regulation of HO-1 expression; pre-treatment with Sch A may exert a protective effect on HT-29 cells by preventing HO-1 overexpression, which may be even more detrimental to the cells.

NF-κB transcription factor plays a critical part in regulating immune and inflammatory responses, as well as controlling cell proliferation and cell death^56^. Furthermore, elevated ROS levels could activate NF-κB signalling through nuclear translocation^57^. NF-κB if it is activated excessively or improperly is undesirable to the host. The ability of DON to activate the NF-κB pathway has been extensively documented in literature^19,21,27,28,52^. In this study, we report that DON activated NF-κB during inflammation. Our immunofluorescent data clearly showed the DON induced translocation of Nrf2 from cytoplasm to nucleus. This result appears to be strongly linked to the mycotoxins’ capacity to trigger ROS production. Sch A reduced DON-induced NF-κB activation by preventing its nuclear translocation. Also, Sch A diminished intracellular ROS, thus affected NF-κB activation and the downstream inflammatory responses.

It is evident that DON activates NF-κB signalling which then triggered various signalling pathways, such as MAPKs that are essential for controlling inflammation by mediating the production of inflammatory factors^19,20^. Therefore, to determine whether the MAPK signalling pathways contributed to the Sch A-induced inhibition of the inflammation, the three MAPKs (JNK, p38 MAPK and ERK) were examined. Our data revealed that the DON-induced phosphorylation of p38 and ERK were markedly suppressed by Sch A pre-treatment. This suggested that Sch A reduced inflammation caused by DON, at least partially, by down-regulating the MAPK signalling pathways, which then blocked NF-κB inactivation.

COX-2 is an immediate early response gene, and is regarded as an inflammatory marker and linked to numerous inflammatory conditions as well as carcinomas^58^. PGE2 is one of the important mediators produced at the inflammatory sites by the COX-2 enzyme. Our results were consistent to previous findings where COX-2 expression was induced following DON exposure^27,28^. However, the failure of induction of PGE2 by DON may be due to the relatively low concentration of DON employed in the current study. Pre-treatment with Sch A considerably diminished COX-2. Sch A also inhibits PGE2 production by down-regulating COX-2 mRNA and protein levels. Sch A appears to down-regulate DON-triggered COX-2 expression partially by affecting the cellular redox status and NF-κB activation. Sch A may also control COX-2 expression by modulating the Peroxisome proliferator activated receptor γ/Retinoid X receptor (PPARγ/RXR) complex, as demonstrated by other investigators^59^. Additional mechanistic studies are necessary to elucidate these.

In our study, we also examined if the Sch A effects on IL8 mRNA expression and secretion. IL8 is secreted by phagocyte and mesenchymal cells following inflammation, infection, ischemia, trauma, etc^60^. It activates neutrophil chemotaxis and promotes inflammation at the site of infection^61^. Our results showed that DON significantly stimulated IL8 mRNA expression and secretion as in other studies^19,20,22^. It has been proposed that the augmented IL8 level following DON exposure was resulted from the activation of the MAPKs and NF-κB inflammatory pathways^20^. Pre-treatment with Sch A effectively repressed the mRNA and secretion of IL8.

**Figure 12:**
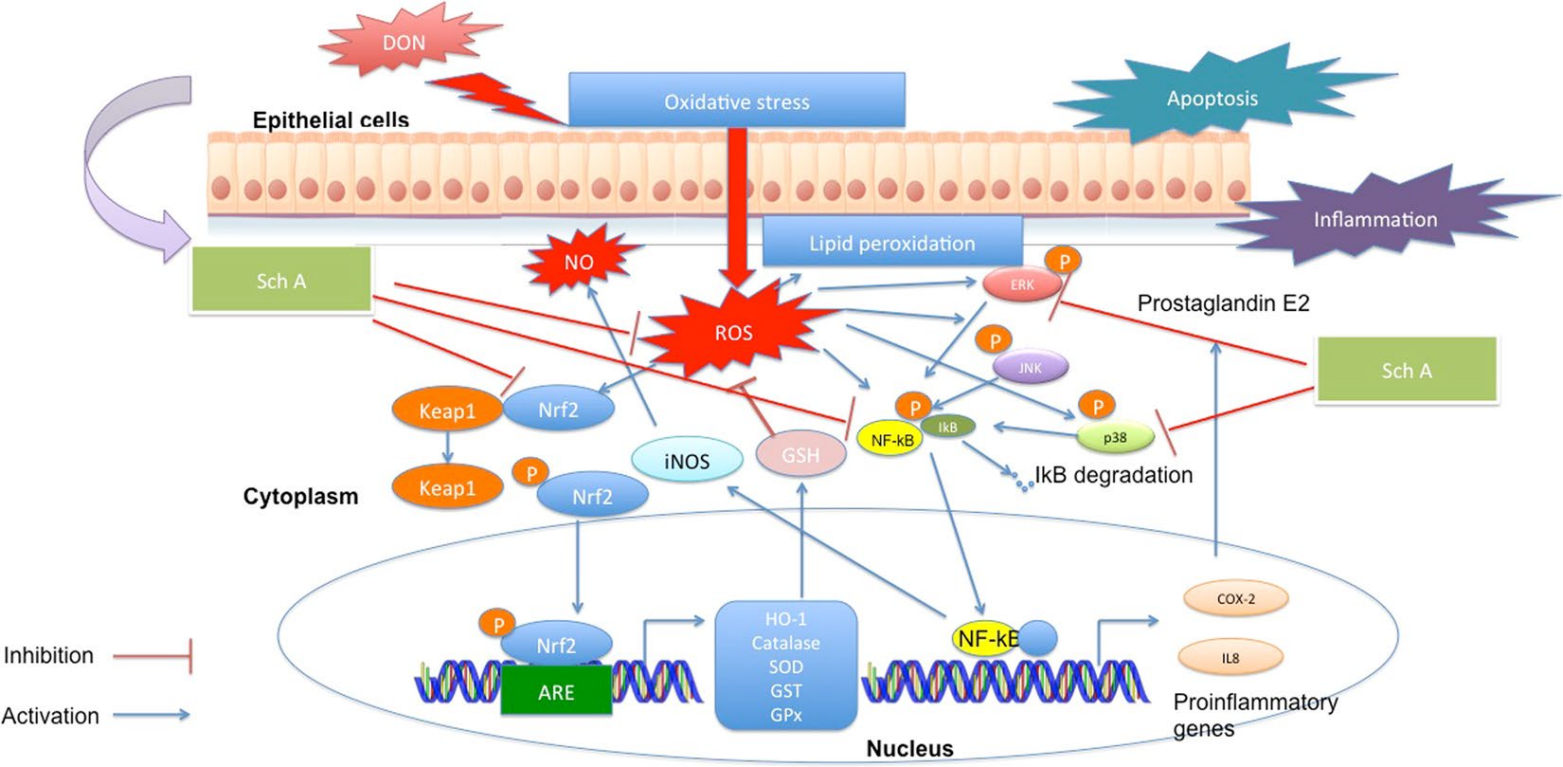
A summary of the cytoprotective mechanisms of Sch A on DON-induced toxicity. Sch A exerts its cytoprotective against DON by protecting the intestinal epithelial cells from cytotoxicity, oxidative damage and inflammation. Such effects were possibly mediated by modulating NF-κB and MAPKs pathways, as well asNrf2/HO-1 signalling.

Over the years, research on dietary antioxidants and their cytoprotective role in preventing DON toxicity has gained enormous popularity. As DON is a frequently occurring food and feed contaminant; it would be of great interest to discover any substances that could be used as potential therapeutic to decrease the toxic effects caused by DON in the human body. Our study suggested that Sch A exerts its cytoprotective effects against DON by protecting the intestinal epithelial cells from cytotoxicity, oxidative damage and inflammation. Such effects were perhaps mediated by NF-κB, MAPKs and Nrf2/HO-1 signalling pathways. A summary of the cytoprotective mechanisms of Sch A on DON-induced toxicity was depicted in Fig. 12. Several *Fusarium* species and toxins such as nivalenol, zearalenone and fumonisins co-occur in wheat and maize^62^, Sch A modulation may have a positive effect on these mycotoxins. Application of modern molecular biology techniques will also help to elucidate the mechanistic pathways regarding the dynamic interaction that occurs between mycotoxins, Sch A and IECs. Although more future studies of the exact mechanisms is necessary, our study has demonstrated that Sch A may be used as a natural bioactive compound with anti-oxidative and anti-inflammatory functions and may be a useful therapeutic approach for oxidative or inflammation-mediated diseases such as chronic intestinal inflammatory diseases like IBD. Despite there are several limitations in this cell-culture approach, these data provide invaluable information for future designs of more comprehensive of animal and human studies.

## Materials and Methods

### Chemicals and reagents

All cell culture reagents were purchased from Gibco-Life Technology (Eggenstein, Germany). Sch A was from MedChem Express (Monmouth Junction, NJ, USA). DON was from Sigma Chemical Company (St. Louis, MO, USA). They were dissolved in dimethylsulfoxide (DMSO) (Sigma) and stored at −20 °C before use. Phosphate buffered saline (PBS), sodium biocarbonate, diethylpyrocarbonate (DEPC) and chloroform were purchased from Sigma. Ammonium persulfate, N, N, N′, N′-tetramethyl-ethane-1,2-diamine (TEMED), acrylamide, resolving gel buffer, stacking gel buffer, 10% (w/v) Tween 20 and 20% (v/v) sodium dodecyl sulfate (SDS) were purchased from Biorad (Richmond, CA, USA). 30% (w/w) hydrogen peroxide (H_2_O_2_) solution, absolute ethanol, and isopropanol were from Merck (Darmstadt, Germany). RNAisoPlus was purchased from Takara (Otsu, Japan). HiScript^TM^ RT SuperMix for qPCR and AceQ qPCR SYBR Green Master Mix were obtained from Vazyme Biotech Co. (Piscataway, NJ, USA).

### Cell line and culture conditions

Human colorectal adenocarcinoma HT-29 cells were maintained at 37 °C in a humidified incubator with 5% CO_2_, using Dulbecco’s modified eagle’s medium (DMEM) supplemented with 10% fetal bovine serum (FBS) (v/v). All cells were tested for mycoplasma contamination with a MycoAlert mycoplasma detection kit (Lonza, Basel, Switzerland) prior to use.

### Cell viability by CCK-8 assay

CCK-8 was carried out to evaluate cell viability following the treatments with different concentrations of DON and Sch A. The assay was carried out according to the manufacturer’s instruction (Dojindo, Kunamoto, Japan). In brief, DON was administered to the cells (1 × 10^4^ cells/well in 96-well culture plates) at a final concentration of 0.25, 0.5 and 1 μM. The concentrations of DON were selected (0.25–1 μM) based on our previous studies^22,63,64^ and are similar to the levels that could be encountered in the gastrointestinal tract of animals or human tissues after ingestion of DON-contaminated food or feed. Assuming that DON ingested in one meal is diluted in 1 liter of gastrointestinal fluid and is totally bioaccessible, the *in vitro* concentrations to be used in this study correspond to food contamination ranging from 150 ng/g to 295 ng/g of DON. Sch A was added to the cells at a final concentration of 2.5, 5 and 10 μM. These concentrations were selected on the basis that they did not affect the cell viability as determined by the CCK assay. Cytoprotective effect of Sch A was studied by exposing cells to Sch A for 24 hours followed by DON for 24 hours. The final concentration of DMSO in all the treatments did not exceed 0.5% v/v. At the end of the treatment, CCK-8 solution (10 μl) was added and incubated at 37 °C for one hour. The optical density (O.D.) was recorded on the Multiskan microplate reader (ThermoFisher Scientific, Waltham, MA, USA) at 450 nm. Cell viability is expressed as the percentage of the mean value normalized to the negative control (untreated cells with 0.5% v/v DMSO).

### Cell cycle analysis

HT-29 cells after 24 hours of Sch A pretreatment were exposed to DON for another 24 hours. The cells were fixed in 70% (v/v) ice cold ethanol and then stained with propidium iodide (PI) solution (PBS with 10 mM Tris base, 10 mM sodium chloride (NaCl), 700 U/L RNase A, 50.1 mg/L PI and 0.1% (v/v) Nonidet P-40) and flow cytometery was performed using a FACSCalibur flow cytometer (BD Biosciences, Franklin Lakes, NJ, USA). Data analyses were done with FlowJo software (Tree Star, Ashland, OR, USA) based on 10,000 cells.

### Determination of apoptosis

For apoptosis experiments, cells following the same procedures as described for cell cycle analysis were stained using the Annexin V apoptosis detection kit from BD Pharmingen (San Jose, CA, USA), and acquired on a FACSCalibur flow cytometer (BD Biosciences), and then analyzed with FlowJo software (Tree Star). Annexin V fluorescein isothiocyanate (FITC)- or PI-positive cells were counted as apopotic cells (either early or late apoptotic), and the remaining cells were designated the surviving cell fraction.

### Estimation of intracellular reactive oxidative species (ROS)

Cells (1 × 10^4^ cells/well) were incubated with 2′,7′-dichlorodihydrofluorescein diacetate (DCF-DA) (0.05 mM) (Sigma) for 30 minutes, followed by incubation with 1 μM DON for 0.5–6 hours. In the Sch A pre-treatment samples, the cells were treated with 2.5–10 μM Sch A for 24 hours and then exposed to DCF-DA for 30 minutes followed by 1 μM DON for 0.5–6 hours. For cells with 24 hours of DON treatment, cells were incubated with DCF-DA for 30 minutes at the end of incubation. The samples were read in a fluorimeter, with excitation and emission wavelengths of 480 nm and 520 nm (Model VICTOR X3, PerkinElmer, Courtaboeuf, France). The values were expressed as % relative fluorescence as compared to the control.

### Thiobarbituric acid reactive substances (TBARS) assay

The malondialdehyde (MDA) content was quantitated as a marker of lipid peroxidation. This assay was based on the reaction between MDA and 2-thiobarbituric acid (TBA) as a thiobarbituric acid reactive substance (TBARS) to form a 1:2 MDA-TBA adduct. Hence, the amount of TBARS correlated with the MDA produced. To obtain the whole cell lysates, cells undergoing the same treatment as described above, were lysed in (radioimmunoprecipitation assay) RIPA buffer. The total protein concentration was determined by the DC protein assay (Biorad) using bovine serum albumin (BSA; GE Healthcare, Chicago, IL, USA) as the standard. 100 μl of whole cell extracts were then incubated with 100 μl of 10% (v/v) trichloroacetic acid (TCA; Sigma) and centrifuged. The resulting supernatants (100 μl) were mixed with 100 μl of 8% (v/v) SDS solution and 1 ml of 0.8% TBA solution in 10% acetic acid (Sigma) and boiled in 95 °C water bath for 1 hour. The mixtures were cooled and centrifuged. The absorbance was measured at 532 nm with the Multiskan microplate spectrophotometer (ThermoFisher Scientific). The concentration of TBARS was calculated using the MDA standard curve and is expressed as μM/mg of protein.

### Nitric oxide (NO) assay

Following the same treatments as described above, the level of NO in the culture supernatants was measured by the NO assay using the Greiss reagent kit (ThermoFisher Scientific, Waltham, MA, USA), which measures the amount of nitrite in the culture supernatants.

### Antioxidant enzymes

Cells following the above-mentioned treatments, were lysed in RIPA buffer as described above to obtain whole cell lysates for the following antioxidant enzyme assays.

Glutathione S transferase (GST) was determined by the method of Habig *et al*., with slight modifications^65^. The assay mixture contained 0.1 M phosphate buffer, pH 6.5, 100 mM 1-chloro-2,4-dinitrobenzene (CDNB; Sigma), 200 mM GSH (Sigma) and 20 μl extracted protein in a final volume of 200 μl. The change in absorbance was measured at 340 nm for 5 minutes at 1-minute intervals in a 96-well plate (Corning, NY, USA) using the Multiskan microplate spectrophotometer (ThermoFisher Scientific). The enzyme activity was calculated based on extinction coefficient of GS-CDNB, E340 = 0.0096 μM^−1^ cm^−1^ and expressed as nmoles CDNB conjugated/minute/mg protein. Catalase (CAT) activity was measured using an Amplex Red Catalase Assay Kit (Molecular Probes, Eugene, OR, USA) according to the manufacturer’s instruction. Intracellular superoxide dismutase (SOD) activity was measured using the SOD Assay Kit-WST (Dojindo). Glutathione (GSH) level was quantified with a GSH/GSSG ratio assay kit (Abnova, Walnut, CA, USA) according to the manufacturer’s instruction. Glutathione peroxidase (GPx) activity was determined as described previously with some modifications^49^. The reaction mixure contained 0.1 M phosphate buffer pH 7.5, 1 mM EDTA and 2 mM sodium azide, 0.1% Triton X-100, 2 mM reduced GSH and 0.2 mM NADPH, 2.5 U freshly prepared glutathione reductase (GR) and 0.25 mM H_2_O_2_. Twenty microliter of cell extracts were added to 200 μl of reaction mixture. One unit of GPx will reduce 1 μmol of glutathione disulfide (GSSG) per minute at pH 7.5. Assays were conducted at 25 °C during 5 minutes. GPx enzymatic activity was calculated by using the molar absorptivity of NADPH (6.22 mM^−1^ cm^−1^) and expressed as μmol of NADPH oxidized/minute/mg of protein.

### Measurement of interleukin 8 (IL8) and prostaglandin E2 (PGE2)

Following the same treatments as described above, the level of IL8 in the culture supernatants was determined using IL8 human ELISA kit (ThermoFisher Scientific); and the PGE2 level was determined using PGE2 monoclonal enzyme immunoassay (EIA) kit (Cayman Chemicals, Ann Arbor, MI, USA) according to the manufacturer’s instructions.

### Real-time quantitative polymerase chain reaction (qPCR)

HT-29 cells were treated as described above. Total RNA was extraction using RNAiso Plus following the manufacturer’s protocol. Total RNA (500 ng) was converted to cDNA using the HiScript^TM^ RT SuperMix for qPCR. qPCR was perfomed on a StepOnePlus Real-Time PCR system (Applied Biosystems, Foster City, CA) using AceQ qPCR SYBR Green Master Mix to measure the mRNA expression levels of the downstream gene of Nrf2 signalling pathway, heme oxygenase (HO-1). Furthermore, COX-2, a key enzyme in production of PGE2, together with inflammatory cytokines, including IL8 were also measured. Assessment of glyceraldehyde-3-phosphate dehydrogenase (GAPDH) levels was also performed which served as an internal control for RNA integrity and loading^66,67^. GAPDH amount were indifferent between all treatment groups investigated (data not shown). Human nucleotide sequences of the primers were shown in Table 1. Samples were thermocycled using the default fast program (45 cycles of 95 °C for 5 s and 60 °C for 30 s). The amplification specificity was checked by both melting curve analysis and gel electrophoresis. Relative changes in gene expression levels in cultured intestinal cells were analyzed using the 2^−^△△^CT^ method as described previously^68^.

**Table 1:** Primer sequences used for qPCR.

Genes	Primers	Forward (5′→3′)	Reverse (5′→3′)	Accession number
Heme oxygenase	HO-1	GCCACCAAGTTCAAGCAGCT	CAGTGCCCACGGTAAGGAAG	NM_002133.2
Cyclo-oxygenase-2	COX-2	CAGCACTTCACGCATCAGTT	CGCAGTTTACGCTGTCTAGC	NM_000963.3
Interleukin 8	IL8	CTGATTTCTGCAGCTCTGTG	GGGTGGAAAGGTTTGGAGTATG	NM_000584.2
Glyceraldehyde-3-phosphate dehydrogenase	GADPH	CATGTTCGTCATGGGGTGAACCA	AGTGATGGCATGGACTGTGGTCAT	NM_001289745.2

### Protein extraction, SDS-PAGE and immunoblotting

The cells were pre-treated with Sch A for 24 hours, followed by incubation of DON for the indicated periods. Cells were then extracted for either total proteins (whole cell extracts) or detergent-insoluble protein fractions as reported previously^69^. The whole cell extracts were obtained by lysing cells in RIPA lysis buffer, supplemented with protease inhibitor cocktail (Sigma) and phosphatase inhibitors (Cell Signalling, Beverly, MA, USA). Nuclear and cytoplasmic proteins were using the nuclear extraction kit (Abcam, Cambridge, MA, USA) following the manufacturer’s instructions. Protein concentration was measured by the DC protein assay (BioRad).

Proteins (10–50 µg) were separated by electrophoresis (SDS-PAGE), and then transferred onto a polyvinylidene difluoride (PVDF) membrane (Millipore, Darmstadt, Germany). Blots was blocked with 5% BSA in Tris-buffered saline (TBS) containing 0.05% (v/v) Tween 20 (TBST) buffer. Blots were probed with diluted rabbit primary antibodies from Cell Signalling (1:1000) for HO-1 (#5853), COX-2 (#12282), NF-κB p65 (#8242), p44/42 MAPK (Erk1/2) (#4695), phospho-p44/42 MAPK (Erk1/2) (#4370), JNK2 (#9258), phospho-SAPK/JNK (#4668), p38 MAPK (#8690), phospho-p38 MAPK (#4511), histone H3 (#4499 S), β-Actin (#4967) or GAPDH (ab181602; Abcam), followed by horseradish peroxidase (HRP)-conjugated anti-rabbit IgG (#170–6515, BioRad) secondary antibodies. The blots were developed using Clarity Western ECL blotting kit (BioRad) and chemiluminescence was detected with a digital imaging system (ChemiDoc XRS + system with image lab software, BioRad). Quantification was obtained by ImageJ software (Ver. 1.48, National Institutes of Health, MD, USA)^70^.

### Immunofluorescence

Nuclear localization of Nrf2 was detected by immunofluorescence confocal microscopy. Cells were exposed to DON at 1 μM for 3 hours for Nrf2. The duration of DON treatment for Nrf2 was determined based on our preliminary experiments (data not shown). For the Sch A pre-treated samples, 10 μM Sch A exposure was carried out for 24 hours, followed by DON exposure at 1 μM for 3 hours. Cells were also exposed to 10 μM Sch A alone for 24 hours. After treatment, cells were fixed with 10% (v/v) formalin and ice cold absolute methanol, followed by permeabilization with 0.1% Triton X-100. They were then incubated with Nrf2 primary antibody (1:500 dilution) (#12721; Cell Signalling) and goat anti-rabbit IgG H&L (Alexa Fluor® 488) (ab150077, Abcam) as secondary antibody (1:200), and subsequently Hoechst staining and viewed and analyzed in a Zeiss LSM 710 NLO confocal microscope (Carl Zeiss, Thornwood, NY, USA).

### Statistical analyses

All assays were expressed as mean ± standard error of mean (SEM) for the number of separate experiments indicated. Data analyses were performed using the GraphPad PRISM 6.0 software (Graphpad Software Inc., San Diego, CA). All data were first evaluated for normality with the Shapiro–Wilk and Levene’s variance homogeneity test. One-way analysis of variance (ANOVA) with the Kruskal-Wallis test, followed by the Mann-Whitney U test was used to analyze non-parametric data. One-way ANOVA with Tukey’s multiple comparison test was used for the analysis of parametric data. Data are significantly different at *p* < 0.05, according to the post hoc ANOVA statistical analysis.
